# Supplementary material for: ProteoMeter: a pipeline for integrating multi-PTM and limited proteolysis data to reveal modification-structure coupling at the residue level
Source: NAR Genom Bioinform. 2026 Jul 14;8(3):lqag073. doi: 10.1093/nargab/lqag073 (PMC13365959; doi:10.1093/nargab/lqag073)
Supplement: lqag073_Supplemental_File [file lqag073_supplemental_file.pdf]

Supplemental Text

April 29, 2026

1 Methods

1.1 Experiment details

Viruses and Viral Titration. Wild-type HCoV-229E was obtained from BEI resources for research purposes (NIAID, NIH: Human Coronavirus, 229E, NR-52726). Viral stocks were first generated (p1) in MRC5 (human lung fibroblast) and then p2 stocks were generated in Huh7 (liver epithelial) cells. Huh7 cells were also used to quantify viral titers following infection. Viral titers were quantified using standard plaque assay approaches [Sims et al., 2005]. Briefly, cells (4.5×10^5 cells per well) were plated in 6-well dishes from Corning (Cat# 08-757-214). Viral samples were serially diluted, plated, and grown under 0.8% agarose and media on confluent monolayers. Plaques were visualized and counted following neutral red staining.

Immortalized cell culture. A549 (human lung epithelial cells, BEI resources: NIAID, NIH: Human Lung Carcinoma Cells (A549) Expressing Human Angiotensin-Converting Enzyme 2 (HA-FLAG), NR-53522) cells were cultured in high glucose Dulbecco's modified essential medium (DMEM; Gibco 11-995-073), containing 10% fetal bovine serum (FBS; Cytiva; SH3007003HI) and 1% Antibiotic-Antimycotic 100X (Anti-Anti; Gibco). MRC5 (human lung fibroblasts, ATCC CCL-171), were cultured in alpha minimum essential medium (α-MEM; Gibco 12-561-056) containing 10% FBS and 1% Anti-Anti. HuH7 (human liver epithelial cells, Japanese Cancer Resource Bank JCRB0403) were cultured in low glucose DMEM (Gibco 11-885-092) containing 10% FBS and 1% Anti-Anti. Media serum concentrations were reduced to 4% prior to infection.

Sample Processing for Proteomics. A549 (PTM studies only) and MRC5 (PTM and LiP studies) cells were mock-infected or infected with HCoV-229E (MOI 3) and harvested at 8, 16, or 24 h post infection. Media from infected wells was collected at 8, 16, and 24 h post infection to determine HCoV-229E growth kinetics in both cell types. For LiP analysis, whole cell pellets were snap frozen and stored at -80 °C prior to being lysed using a hand-operated pellet-pestle homogenizer inside a biological safety cabinet. After measuring protein concentration through BCA, the lysates were subjected to LiP-MS. For PTM analysis, cells were washed once with 1× phosphate buffered saline (PBS) and then washed again with PBS containing 100mM NEM for replicates 1 through

4. Replicate 5 for each condition (the total thiol sample) the final wash was performed with 1× PBS alone. Washes were removed and cells were frozen in their plates and stored at −80°C before harvest.

Sample processing for multi-PTM proteomics. Plates containing infected or mock MRC5 were lysed on ice with 200µL of 250 mM MES (pH 6.0) containing 5% SDS, 1% Triton X-100 with or without 100 mM NEM. Cells were collected and incubated in dark for 30min. DNA was sheered using a probe sonicator (Fisher Scientific Series 60 Sonic Dismembrator). Cell debris was removed by centrifugation at 13.000 g, 4 °C for 10 min. All samples were incubated at 55 °C, 850 rpm for 30 min. Automated sample cleanup and protein digestion was performed as described previously [Gluth et al., 2024, Leutert et al., 2019]. Briefly, the volumes of all samples in deep-well plates were adjusted to 500 µL, and 500 µL absolute ethanol was added (sample/binding plate). The magnetic beads were conditioned and aliquoted into a bead plate with a protein-to-bead ratio (w/w) of 1:5. Three wash plates contained 1 mL of 80% ethanol/well. The comb, bead, sample/binding and wash plates were placed onto KingFisher Flex (Thermo Scientific). The R2-P1 program was used to transfer beads to sample/binding plate, incubate for binding, and wash protein-bound beads for three times. The beads with proteins were transferred to a digestion plate containing 500 µL of digestion cocktail/well (1:100 trypsin and 1:100 Lys-C in 50 mL HEPES buffer, pH 7.7). Then digestion was conducted on a ThermoMixer at 37 °C, 850 rpm for 3h. The digested samples (the first elution) were collected on a MagnaBot FLEX 96 magnetic plate (Promega). 500 µL of 250 mL HEPES with 5% ACN (pH 7.7) per well was used to wash the beads and collected (the second elution buffer). Two elution was combined. Cleaned peptide samples were labeled with TMTpro 18-plex (Thermo Scientific) by incubation at room temperature, 850 rpm for 1h, followed by quenching with hydroxylamine. The TMT:peptide ratio (w/w) was 2.5:1. The labeled samples were cleaned up by C18 SPE desalting and aliquoted for global and redox, phosphorylation, and acetylation enrichment. Peptide level resin-assisted capture (RAC) of redox-modified peptides was conducted following the procedures published elsewhere using the thiol-affinity resin made in-house [Day et al., 2022, Guo et al., 2013, Gaffrey et al., 2021]. Global and redox peptide samples were fractionated on a nanoAcquity LC system (Waters) using a reversed-phase LC column (65 cm × 200 µm internal diameter packed with 3 µm Phenomenex Jupiter C18 particles) for 12 fractions [Li et al., 2021]. Automated IMAC phosphopeptide enrichment was conducted on an Agilent Bravo system as described by Abelin et al [Abelin et al., 2023]. The flowthrough from IMAC were collected and used for acetylation enrichment using PTMScan HS Acetyl-Lysine Motif [Ac-K] kit (Cell Signaling Technologies #46784) on KingFisher as previously described [Gluth et al., 2024]. All final peptide samples were analyzed on an ACQUITY UPLC (Waters) coupled with Q Exactive Plus mass spectrometer (Thermo Scientific) [Gluth et al., 2024]. Raw spectra were processed using MSGF+ (10.1038/ncomms6277) for PTM proteomics data. *Limited proteolysis-based mass spectrometry (LiP-MS).* The MRC5 cell pellets were lysed with three cycles of freeze-thawing, and protein concentration in the lysates were measured

using BCA assay. Each sample was divided into two parts- the control and the LiP sample- each containing equal protein amounts. The LiP sample was treated with non-specific protease, Proteinase K at an enzyme:substrate ratio 1:200. The control samples were received an equivalent volume of PBS. All the samples were incubated at 25 °C for 1 min, followed by heat inactivation at 98 °C for 5 min. The proteins were denatured by adding solid urea to achieve an 8 M solution. Subsequently, the samples were reduced with DTT (5 µM), alkylated with IAA (40 µM), and subjected to overnight digestion with LysC (enzyme:substrate = 1:100), and trypsin (enzyme:substrate = 1:100). Samples were acidified to 1% FA and then were desalted with C18 solid phase extraction. The final peptide concentrations were quantified using BCA assay, and peptide mixtures were analyzed using an Orbitrap Exploris mass spectrometer. Raw spectra were processed using FragPipe (10.1016/j.mcpro.2021.100077) for LiP-MS data. FragPipe was run using the following settings for trypsin-only (single-digest) samples: Dynamic Mods: M (+15.9949), Protein N-Term (+42.010567), Static Mods: C (+57.021465), Parent ion tolerance = 10ppm, Fully tryptic search, DIA analysis using a spectrum library generated at runtime, digest 500 to 8000 Da, Precursor charge 1+ to 4+. FragPipe was run using the following settings for double-digested samples: Dynamic Mods: M (+15.9949), Protein N-Term (+42.010567), Static Mods: C (+57.021465), Parent ion tolerance = 10ppm, Partially tryptic search, DIA analysis using a spectrum library generated at runtime, digest 500 to 8000 Da, Precursor charge 1+ to 4+. All FragPipe parameters were set to default unless otherwise specified.

2 ProteoMeter Processing

Input data and annotated configuration files used in this work can be found at `https://github.com/PNNL-Predictive-Phenomics/ProteoMeter/tree/main/demo_data`. These are ingested by the script found at `https://github.com/PNNL-Predictive-Phenomics/ProteoMeter/blob/main/demonstration.py`. See the Usage Information Section in this document for further information. The API documentation is available at `https://pnnl-predictive-phenomics.github.io/ProteoMeter/`.

3 Quality Control and Batch Correction

Figure S1 depicts the correlation between LiP samples at the peptide and protein level prior to further processing. Based on these plots, we removed the 24 hour post-infection mock-infected replicate number one and the 24 hour post-infection mock-infected replicate number one as outliers. We then recreated the correlation plots with these outliers placed in the last two rows and columns. Figure S2 depicts analogous plots for the PTM samples. No PTM samples were removed as outliers. However, we note pronounced batch effects in the correlation plots. We performed row-mean centering batch correction and compared

PCA biplots before and after batch correction (Figure S3) to confirm that the dominant batch effect was successfully removed.

4 Abundance and Batch Correction

Abundance correction is generally recommended to help decompose effects of changing protein abundance from changes in the fraction of protein in a modified state and to reduce noise. We implement two methods for abundance correction: paired and statistical. In the case of the experiments presented here, global proteomics data and PTM/LiP data are drawn from the same samples (i.e., they are paired), allowing for direct abundance sample-by-sample abundance correction. When samples are not paired, or when specified by the user, we compute protein abundance correction using the mean fold change across samples for those proteins having a statistically significant log2 fold-change in abundance between treatment and control (at a configurable significance threshold that defaults to $p < 0.05$).

5 Notes on Missingness

During the quality control step, a user-defined threshold for an acceptable number of missing values per condition is used. If this threshold is exceeded for a given protein, the measurements of that protein are discarded for the condition. Different roll-up methods treat missing values differently; for "sum" method the missing values are treated as zero intensity while for "median" and "log2mean" methods the missing values are ignored. After that, the missing values are handled using the same approach as in standard proteomics data analysis, which is to ignore missing values when calculating fold changes and p-values. The number of missing values is reported in the output for reference.

6 Notes on Cut Site Quantification

To roll up to the cleavage site, the intensities of all observed peptides sharing a ProK cleavage site (after abundance correction, missingness filtering, and batch correction) are aggregated by a user-specified aggregation function. Currently, the user may aggregate by averaging in linear intensity space (the default) or in log2 intensity space, or by taking the median intensity. We have selected a default aggregation method similar to that of [Manriquez-Sandoval et al., 2024], which has been validated experimentally. In upcoming work, we perform a systematic *in silico* evaluation of various roll up aggregation functions. As outlined in the discussion of the main text, we caution that such aggregation methods necessarily neglect instrument-specific ion sensitivities, which may introduce errors. We therefore recommend that cut site quantification should be assessed for consistency with peptide-level quantification.

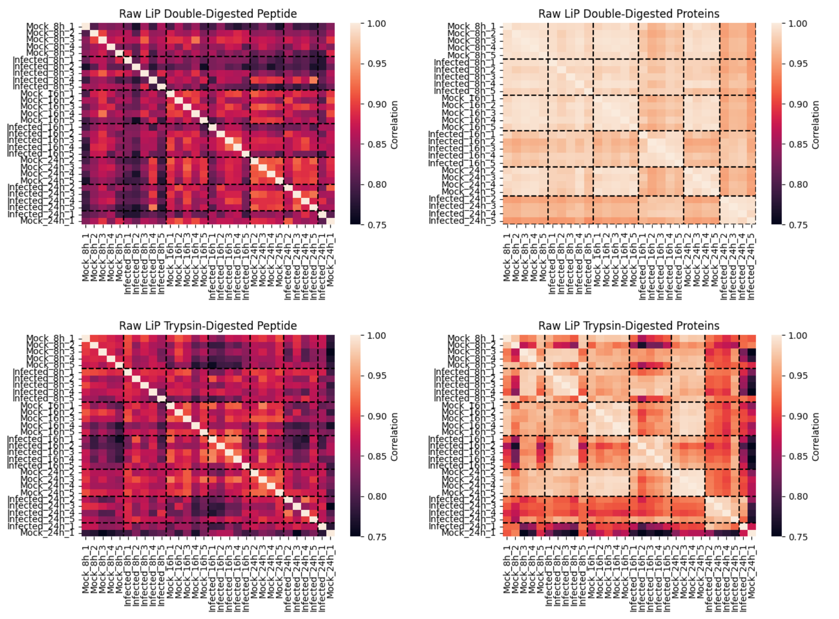

Figure S1: Pearson correlation plots for raw (input) LiP data at the protein and peptide level. Dotted lines separate replicate groups. The last two rows and columns are removed outliers.

7 Notes on Multiple Testing Correction

In PTM experiments, it is common practice to correct for multiple hypothesis testing using the Benjamini-Hochberg procedure at the proteome level. That is, quantification of each PTM site on each protein is treated as a separate test with a total number of tests equal to the total number of captured PTM sites. This is typically done for each PTM type separately. In LiP experiments, however, the relatively high noise often requires an alternative approach. Typically, to obtain high-confidence quantification of changes in exposure, proteins of interest are selected and the Benjamini-Hochberg procedure is performed for each protein individually. The rationale for this is that each tryptic peptide presents an opportunity to reject the null hypothesis that the protein has not undergone a change in its solvent accessibility. This "protein-wise" approach, though widespread, is much less conservative than the multiple hypothesis testing correction typically applied in PTM studies. Caution is required when examining significant LiP changes at the proteome-level. ProteoMeter provides options for conducting both the standard PTM "proteome-wide" and the standard LiP "protein-wise" false discovery control.

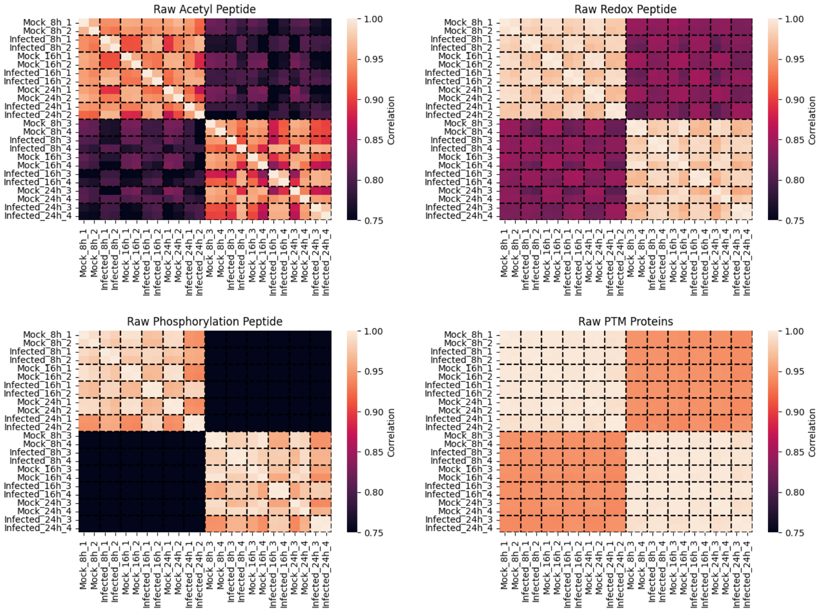

Figure S2: Pearson correlation plots for raw (input) PTM data at the protein and peptide level. Dotted lines separate same-batch replicate groups. Rows and columns are arranged so that the samples from the same batch are grouped together (there are two equally-sized batches). No samples were removed as outliers.

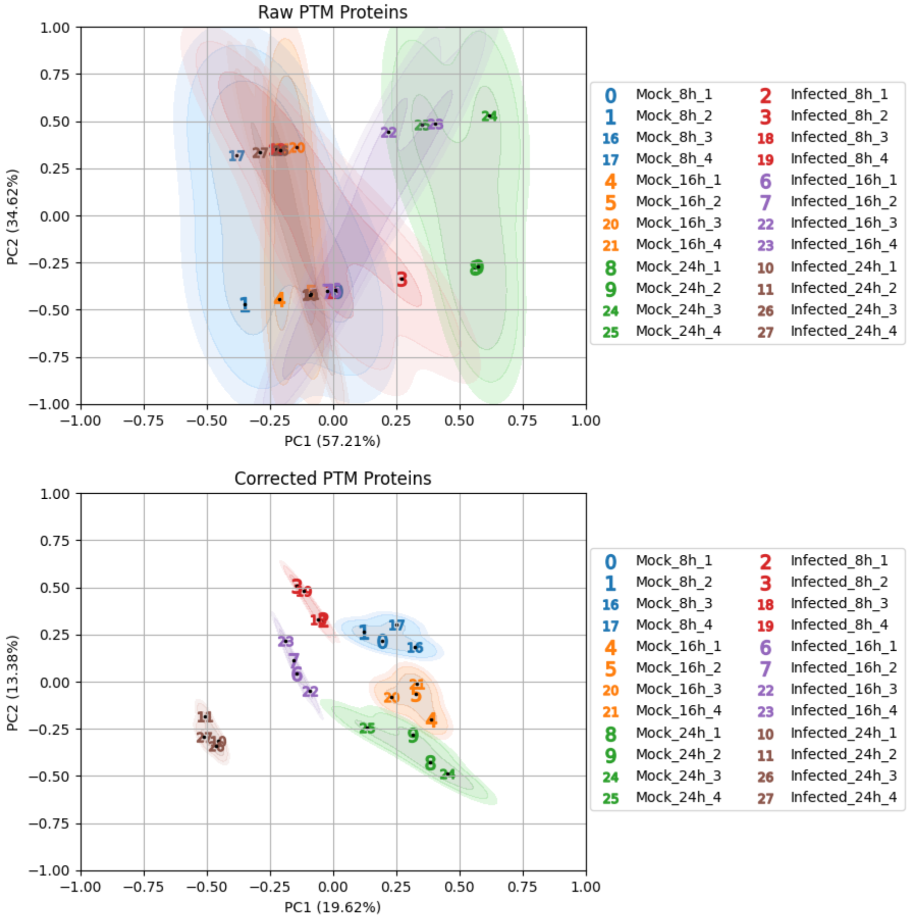

Figure S3: Comparison of PTM PCA biplots (protein-level) before (raw) and after (corrected) normalization and batch correction.

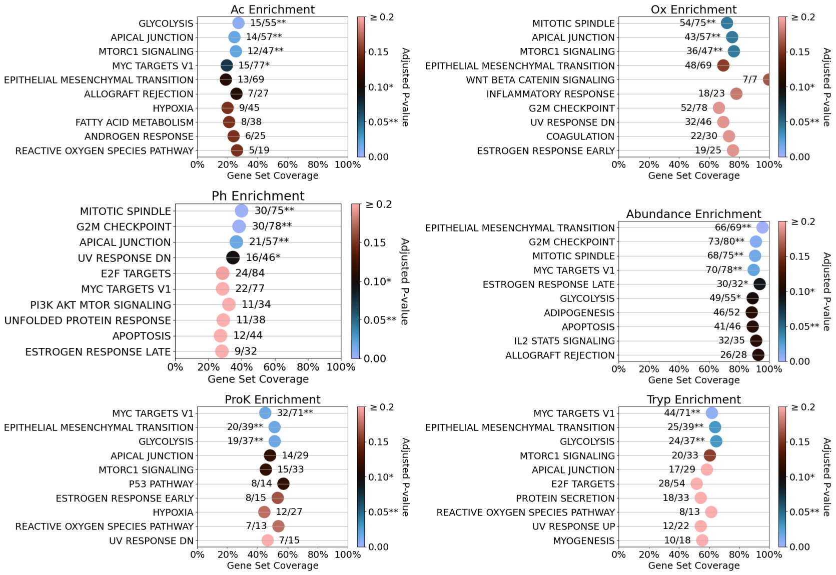

Figure S4: Enrichment analysis across all time points (aggregated) for each PTM type, global abundance changes, and LiP at the ProK cleavage site (ProK) or tryptic peptide (Tryp) level.

8 Enrichment

In this section, we provide additional enrichment analysis plots. Enrichment was performed using GSEAPy on proteins with significant changes in PTMs ($q < 0.05$) or in ProK cleavage site or tryptic peptide quantification ($p < 0.05$). We used human MSigDB Hallmark gene sets for enrichment and computed adjusted p-values using the hypergeometric test.

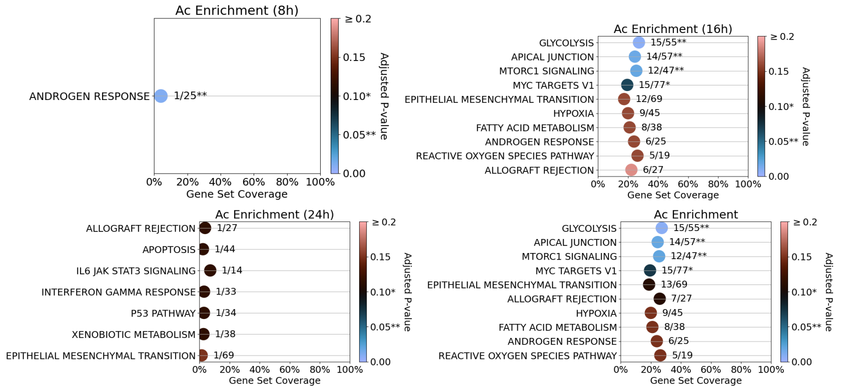

Figure S5: Enrichment analysis for LYS Acetylation at each time point and across all time points.

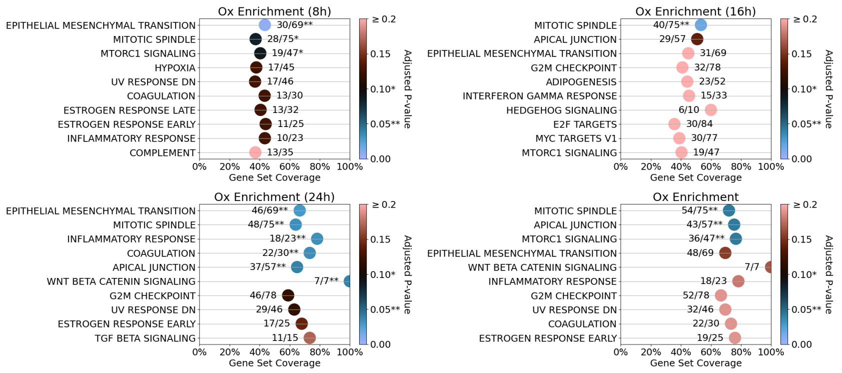

Figure S6: Enrichment analysis for CYS Oxidation at each time point and across all time points.

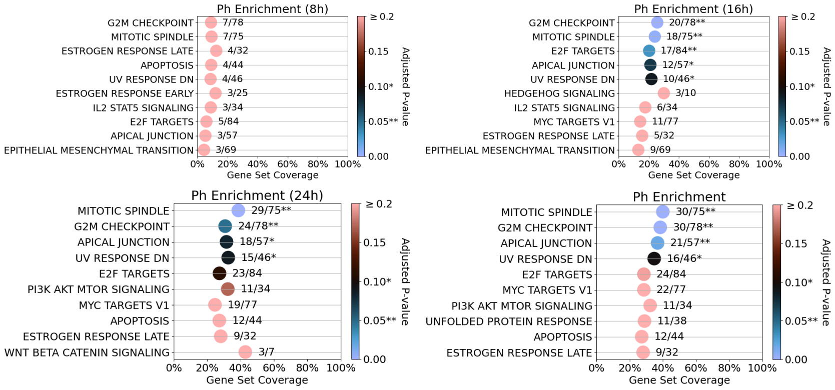

Figure S7: Enrichment analysis for SER/THR Phosphorylation at each time point and across all time points.

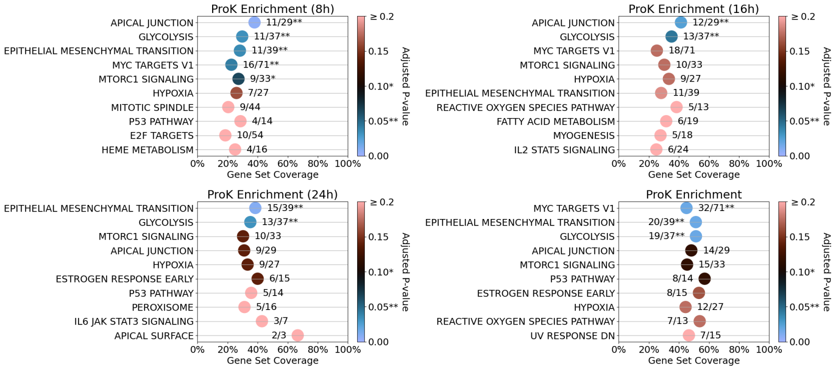

Figure S8: Enrichment analysis for ProK cleavage site signal changes at each time point and across all time points.

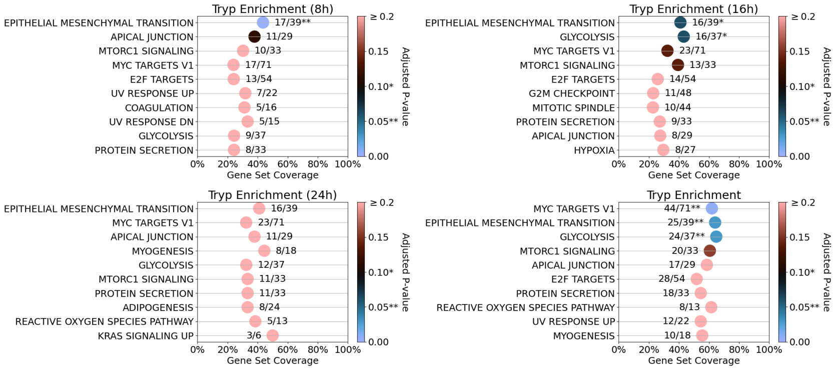

Figure S9: Enrichment analysis for tryptic peptide signal changes at each time point and across all time points.

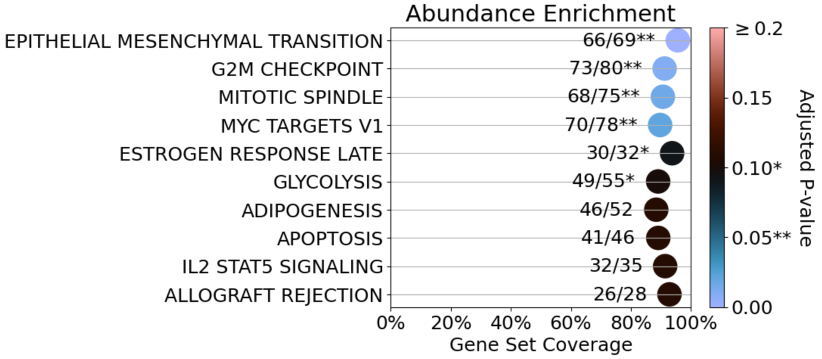

Figure S10: Enrichment analysis for protein abundance changes across all time points.

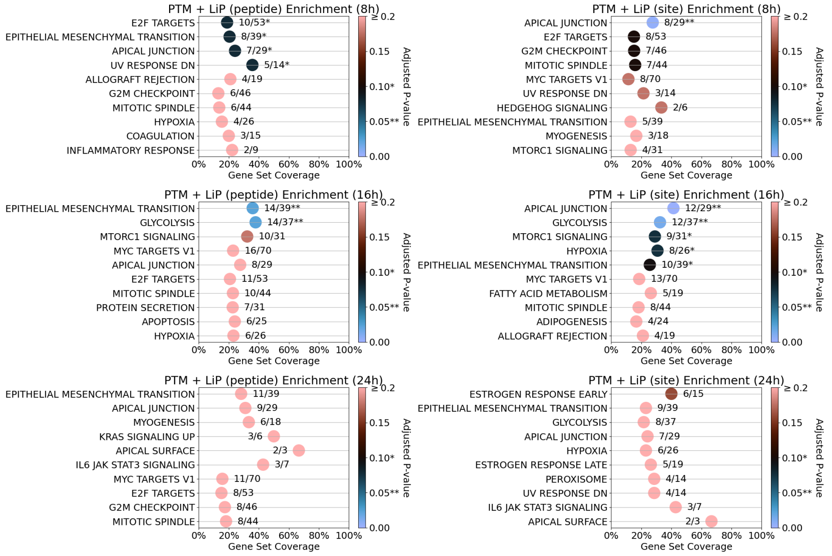

Figure S11: Enrichment analysis for combined PTM and ProK signal changes (site) or combined PTM and tryptic peptide (peptide) changes at each time point.

9 Usage Information

Detailed usage information and example code is available at `https://github.com/PNNL-Predictive-Phenomics/ProteoMeter`. Full API documentation is available at `https://pnnl-predictive-phenomics.github.io/ProteoMeter/`.

9.1 Installation

Source code is available at `https://github.com/PNNL-Predictive-Phenomics/ProteoMeter`. ProteoMeter is installable from PyPI via "pip install proteometer" or similar commands.

9.2 Required Inputs

Example data and configuration files are included with the source code. The file names corresponding to each input are configurable; here we use the default names.

9.2.1 Configuration and Metadata Files

- metadata.tsv
  - Defines the group ID, replicate number, condition (treatment vs control), treatment type, and other variables (e.g., sample time or strain).
- config.toml (lip.toml and/or ptm.toml in the demo_data example)
  - Specifies experiment conditions that affect processing and determines which statistics to compute. See the commented example configs in demo_data for detailed information.
- reference_proteome.fasta
  - Fasta file containing sequence information for the proteome.

9.2.2 LiP Data

- lip_pept.tsv: quantification of peptides after limited proteolysis followed by trypsin digestion (ProK + Trypsin)
- trypsin_pept.tsv: quantification of peptides after trypsin digestion without limited proteolysis (Trypsin only)
- trypsin_prot.tsv: quantification of peptides after trypsin digestion without limited proteolysis (Trypsin only)

In the demo_data/LiP directory of the ProteoMeter repository, we have also included lip_prot.tsv, which contains quantification of proteins after limited proteolysis followed by trypsin digestion. This file is not required, but may be used in place of trypsin_prot.tsv (generally this is not recommended as the abundance quantification will likely be more robust for trypsin-digestion than for limited proteolysis followed by trypsin digestion).

9.2.3 PTM Data

- ptm_pept.tsv: quantification of peptides for a given PTM type
- ptm_prot.tsv: quantification of proteins derived from the peptide-level data

The files above correspond to a specific ptm type (e.g., LYS acetylation). Multiple such files can be provided in a single run. In the demo_data example, we simultaneously analyze LYS acentylaion (acetyl_pept.tsv and acetyl_prot.tsv), CYS oxidation (redox_pept.tsv and redox_prot.tsv), and SER/THR phosphorylation (phospho_pept.tsv and phospho_prot.tsv).

9.3 Outputs

ProteoMeter generates pandas DataFrame objects. In our demonstration, we have saved these outputs to CSV files, each of which corresponds to a data frame generated by the ProteoMeter lip_analysis or ptm_analysis functions. In all output files, fold changes are given in a base 2 logarithmic scale.

- lip_processed_pept.csv: LiP double-digested (ProK + Trypsin) peptide-level data. This includes fold-changes for all peptides, tryptic or otherwise (tryptic peptides are indicated by the pept_type column.)
- lip_processed_prot.csv: Global abundance information used for abundance correction of peptide changes. This includes abundance quantification, fold-changes and statistical significance.
- lip_processed_site.csv: Digestion cut site quantification, fold-changes and statistical significance. Note that each peptide corresponds to two cut sites, but that overlap between the endpoints of peptides is expected, so the number of cut sites is bounded between the number of peptides and twice the number of peptides.
- ptm_processed_site.csv: PTM site quantification, fold-changes and statistical significance.
- ptm_processed_prot.csv: Global abundance information used for abundance correction of PTM changes. This includes abundance quantification, fold-changes and statistical significance.

References

[Abelin et al., 2023] Abelin, J. G., Bergstrom, E. J., Rivera, K. D., Taylor, H. B., Klaeger, S., Xu, C., Verzani, E. K., Jackson White, C., Woldemichael, H. B., Virshup, M., Olive, M. E., Maynard, M., Vartany, S. A., Allen, J. D., Phulphagar, K., Harry Kane, M., Rachimi, S., Mani, D. R., Gillette, M. A., Satpathy, S., Clauser, K. R., Udeshi, N. D., and Carr, S. A. (2023). Workflow enabling deepscale immunopeptidome, proteome, ubiquitylome, phosphoproteome, and acetylome analyses of sample-limited tissues. *Nature Communications*, 14(1).

[Day et al., 2022] Day, N. J., Zhang, T., Gaffrey, M. J., Zhao, R., Fillmore, T. L., Moore, R. J., Rodney, G. G., and Qian, W.-J. (2022). A deep redox proteome profiling workflow and its application to skeletal muscle of a duchenne muscular dystrophy model. *Free Radical Biology and Medicine*, 193:373–384.

[Gaffrey et al., 2021] Gaffrey, M. J., Day, N. J., Li, X., and Qian, W.-J. (2021). Resin-assisted capture coupled with isobaric tandem mass tag labeling for multiplexed quantification of protein thiol oxidation. *Journal of Visualized Experiments*, (172).

[Gluth et al., 2024] Gluth, A., Li, X., Gritsenko, M. A., Gaffrey, M. J., Kim, D. N., Lalli, P. M., Chu, R. K., Day, N. J., Sagendorf, T. J., Monroe, M. E., Feng, S., Liu, T., Yang, B., Qian, W.-J., and Zhang, T. (2024). Integrative multi-ptm proteomics reveals dynamic global, redox, phosphorylation, and acetylation regulation in cytokine-treated pancreatic beta cells. *Molecular & Cellular Proteomics*, 23(12):100881.

[Guo et al., 2013] Guo, J., Gaffrey, M. J., Su, D., Liu, T., Camp, D. G., Smith, R. D., and Qian, W.-J. (2013). Resin-assisted enrichment of thiols as a general strategy for proteomic profiling of cysteine-based reversible modifications. *Nature Protocols*, 9(1):64–75.

[Leutert et al., 2019] Leutert, M., Rodríguez-Mias, R. A., Fukuda, N. K., and Villén, J. (2019). R2-p2 rapid-robotic phosphoproteomics enables multidimensional cell signaling studies. *Molecular Systems Biology*, 15(12):e9021.

[Li et al., 2021] Li, X., Day, N. J., Feng, S., Gaffrey, M. J., Lin, T.-D., Paurus, V. L., Monroe, M. E., Moore, R. J., Yang, B., Xian, M., and Qian, W.-J. (2021). Mass spectrometry-based direct detection of multiple types of protein thiol modifications in pancreatic beta cells under endoplasmic reticulum stress. *Redox Biology*, 46:102111.

[Manriquez-Sandoval et al., 2024] Manriquez-Sandoval, E., Brewer, J., Lule, G., Lopez, S., and Fried, S. D. (2024). Flippr: A processor for limited proteolysis (lip) mass spectrometry data sets built on fragpipe. *Journal of Proteome Research*, 23(7):2332–2342.

[Sims et al., 2005] Sims, A. C., Baric, R. S., Yount, B., Burkett, S. E., Collins, P. L., and Pickles, R. J. (2005). Severe acute respiratory syndrome coronavirus infection of human ciliated airway epithelia: Role of ciliated cells in viral spread in the conducting airways of the lungs. *Journal of Virology*, 79(24):15511–15524.
